# JMJD6-mediated epigenetic silencing of innate immunity promotes pseudorabies virus replication

**DOI:** 10.1128/jvi.00028-26

**Published:** 2026-04-29

**Authors:** Sheng-Li Ming, Ya-Jing Chai, Jia-Ming Yang, Jia-You Xing, Shi-Jun Zhang, Sai-Nan He, Qing-Xia Lu, Jiang Wang, Jia-Jia Pan, Wei-Fei Lu, Lei Zeng, Bei-Bei Chu

**Affiliations:** 1 College of Veterinary Medicine, Henan Agricultural University 70573, Zhengzhou, Henan Province, China; 2 Key Laboratory of Animal Biochemistry and Nutrition, Ministry of Agriculture and Rural Affairs, Zhengzhou, Henan Province, China; 3 Key Laboratory of Veterinary Biotechnology of Henan Province, Henan Agricultural University 70573, Zhengzhou, Henan Province, China; 4 State Key Laboratory of Membrane Biology, School of Pharmaceutical Sciences, Tsinghua University 12442, Beijing, China; 5 Institute for Animal Health, Henan Academy of Agricultural Sciences 74728, Zhengzhou, Henan Province, China; 6 Ministry of Education Key Laboratory for Animal Pathogens and Biosafety, Zhengzhou, Henan Province, China; 7 Longhu Advanced Immunization Laboratory, Zhengzhou, Henan Province, China; 8 International Joint Research Center of National Animal Immunology, Henan Agricultural University 70573, Zhengzhou, Henan Province, China; University of Virginia, Charlottesville, Virginia, USA

**Keywords:** JMJD6, pseudorabies virus, epigenetic regulation, cGAS–STING pathway, viral immune evasion, METTL23

## Abstract

Jumonji domain-containing protein 6 (JMJD6) has been implicated in epigenetic regulation. Here, we demonstrated that JMJD6 was upregulated during pseudorabies virus (PRV) infection and critically enhanced viral replication by promoting virion release. Mechanistically, JMJD6 suppressed PRV-induced histone H4K16 acetylation, a modification associated with chromatin relaxation and DNA damage response activation. This epigenetic modulation attenuated the cGAS–STING-mediated innate immune signaling pathway, leading to reduced interferon production and enhanced viral propagation. Furthermore, we identified METTL23 as a nuclear interactor of JMJD6 upon viral infection, revealing a cooperative role between these proteins in facilitating immune evasion. Importantly, administration of the JMJD6-specific inhibitor JMJD6-IN-1 potently activated innate immunity and restricted PRV replication in mice. Our findings unveil a novel epigenetic strategy employed by PRV to evade host antiviral responses and highlight JMJD6 as a potential therapeutic target for combating herpesvirus infections.

**IMPORTANCE:**

The ongoing conflict between viruses and host antiviral defenses is central to viral pathogenesis. Pseudorabies virus (PRV), a highly contagious alphaherpesvirus, causes severe economic losses in the global swine industry and poses an emerging zoonotic threat to humans. This study identifies the epigenetic modulator, the JMJD6, as a critical host factor exploited by PRV to evade antiviral immunity. Our work uncovers a previously unrecognized epigenetic strategy employed by herpesviruses and establishes JMJD6 as a promising target for developing broad-spectrum antivirals against PRV and related pathogenic herpesviruses.

## INTRODUCTION

Pseudorabies virus (PRV), a neurotropic alphaherpesvirus (1), continues to impose a substantial economic burden on the global swine industry and represents an increasing public health concern due to its zoonotic potential (2, 3). In pigs, PRV is the causative agent of Aujeszky’s disease, which manifests with high mortality in piglets and neurological, respiratory, and reproductive disorders in adults (4). Recent human cases of PRV-associated encephalitis underscore the public health implications of this pathogen (5, 6). While modified live vaccines are widely implemented, they fail to elicit sterilizing immunity, allowing the establishment of latency and periodic viral shedding (7–9). This limitation highlights the critical need for novel therapeutic strategies that effectively disrupt the viral life cycle and bolster host antiviral responses.

The innate immune system constitutes the first line of defense against viral invasion, with the cytosolic DNA-sensing cGAS–STING pathway playing a pivotal role in controlling herpesvirus infections (10–12). Upon detection of viral DNA, this pathway triggers a signaling cascade that culminates in the production of type I interferons (IFNs) and the induction of IFN-stimulated genes (ISGs) (13), establishing a robust antiviral state (14, 15). In response, herpesviruses encode numerous immunomodulators and exploit host factors to evade this defense system (10, 16). Emerging evidence indicates that epigenetic regulation, particularly through histone modifications such as acetylation, serves as a key interface of virus–host interactions (17). Acetylation of histone H4 at lysine 16 (H4K16ac) is associated with open chromatin configurations and activation of DNA damage responses, which can potentiate innate immune signaling (18). However, the mechanisms through which viruses manipulate such epigenetic marks to facilitate immune evasion remain poorly defined.

JMJD6 is a multifunctional protein possessing lysyl hydroxylase and demethylase activities, and it plays diverse roles in transcriptional regulation, RNA splicing, and epigenetic remodeling (19, 20). Initially identified as a receptor for phosphatidylserine on apoptotic cells, JMJD6 has since been shown to modify histones and non-histone proteins through demethylation and desumoylation, thereby influencing gene expression programs (21, 22). Furthermore, JMJD6 modulates the DNA damage response in an enzymatic activity-independent manner by reducing H4K16ac levels (23). Although JMJD6 has been implicated in the replication cycles of several RNA viruses (24–26), its function during DNA virus infection, particularly within the context of herpesvirus-induced epigenetic reprogramming and immune evasion, is largely unknown. Given its nuclear localization and established role in regulating gene expression, we hypothesized that JMJD6 might represent a proviral host factor utilized by PRV to subvert antiviral defenses.

In this study, we demonstrate that JMJD6 expression is upregulated during PRV infection and is essential for efficient viral replication. We establish that JMJD6 functions as a negative regulator of H4K16 acetylation, thereby dampening DNA damage sensing and subsequent activation of the cGAS–STING-dependent IFN response. Our findings reveal a previously uncharacterized epigenetic mechanism adopted by PRV to evade host immunity and position JMJD6 as a promising target for the development of broad-spectrum antiviral therapies.

## RESULTS

### JMJD6 is indispensable for PRV replication

**Fig 1:**
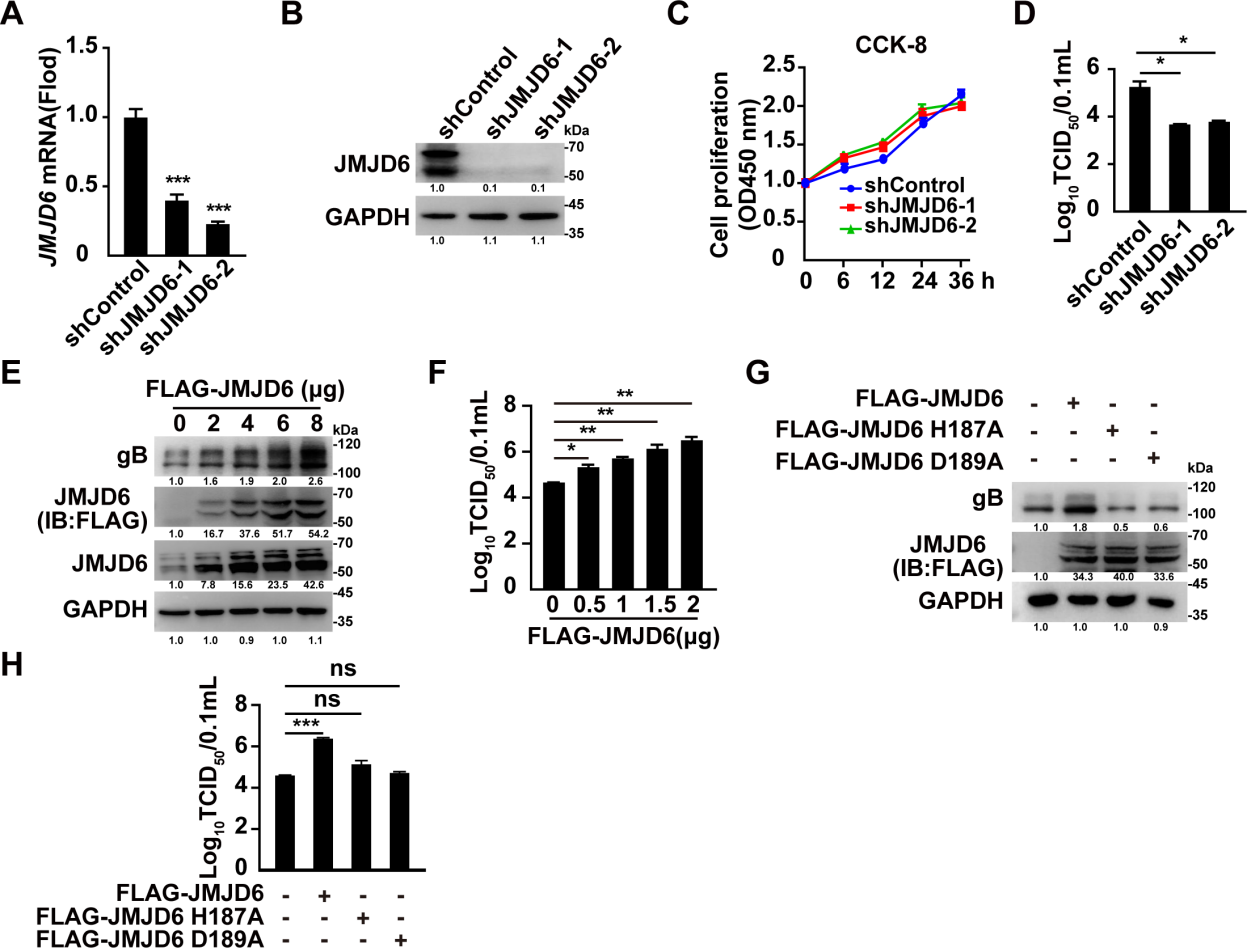
JMJD6 positively regulates PRV replication. (**A and B**) The mRNA (**A**) and protein (**B**) levels of JMJD6 in shControl, shJMJD6-1, and shJMJD6-2 PK-15 cells were analyzed by qRT-PCR and immunoblotting. ***P < 0.001, *n* = 3. (**C**) Cell proliferation was analyzed in shControl, shJMJD6-1, and shJMJD6-2 PK-15 cells for 0–48 h by CCK-8 assay (*n* = 3). (**D**) shControl, shJMJD6-1, and shJMJD6-2 PK-15 cells were infected with PRV-QXX (MOI = 1) for 24 h. The viral titers were analyzed by a TCID_50_ assay. *P < 0.05, *n* = 3. (**E**) PK-15 cells were transfected with FLAG-JMJD6 plasmid (0–8 μg) for 24 h. Cells were then infected with PRV-QXX (MOI = 1) for another 24 h. The expression of PRV gB was analyzed by immunoblotting, *n* = 3. (**F**) PK-15 cells were transfected with FLAG-JMJD6 plasmid (0–2 μg) for 24 h. Cells were then infected with PRV-QXX (MOI = 1) for another 24 h. The viral titers were analyzed by a TCID_50_ assay. *P < 0.05, **P < 0.01; *n* = 3. (**G**) PK-15 cells were transfected with FLAG-JMJD6, FLAG-JMJD6 H187A, or FLAG-JMJD6 D189A plasmid (2 μg) for 24 h. Cells were then infected with PRV-QXX (MOI = 1) for another 24 h. The expression of PRV gB was analyzed by immunoblotting. (**H**) PK-15 cells were treated as in panel F. The viral titers were analyzed by a TCID_50_ assay. ***P < 0.001, *n* = 3. ns, no significance.

To investigate the potential role of JMJD6 in PRV replication, we first knocked down JMJD6 expression in PK-15 cells using RNA interference. Two distinct shRNAs (shJMJD6-1 and shJMJD6-2) significantly reduced both JMJD6 mRNA and protein levels (Fig. 1A and B) without affecting cell proliferation over a 36-h period (Fig. 1C). Upon infection with PRV-QXX, *JMJD6*-knockdown cells produced markedly fewer infectious viral particles compared to shControl-treated cells (Fig. 1D). shJMJD6-2 was selected for subsequent mechanistic studies due to its consistently high knockdown efficiency.

To further examine the involvement of JMJD6 in viral replication, we performed overexpression experiments. Transient transfection of the FLAG-JMJD6 plasmid into PK-15 cells led to a dose-dependent increase in viral yield (Fig. 1E and F). To assess whether the enzymatic activity of JMJD6 is essential for promoting PRV replication, we generated two catalytically inactive mutants, H187A and D189A, based on previous studies (27). As shown in Fig. 1G and H, wild-type JMJD6 markedly enhanced viral gB expression and viral titers, whereas the JMJD6 H187A and D189A mutants failed to exert this effect. Together, these results demonstrate that JMJD6 functions as a proviral host factor and that its enzymatic activity is required for supporting PRV replication.

### PRV infection stimulates JMJD6 expression

**Fig 2:**
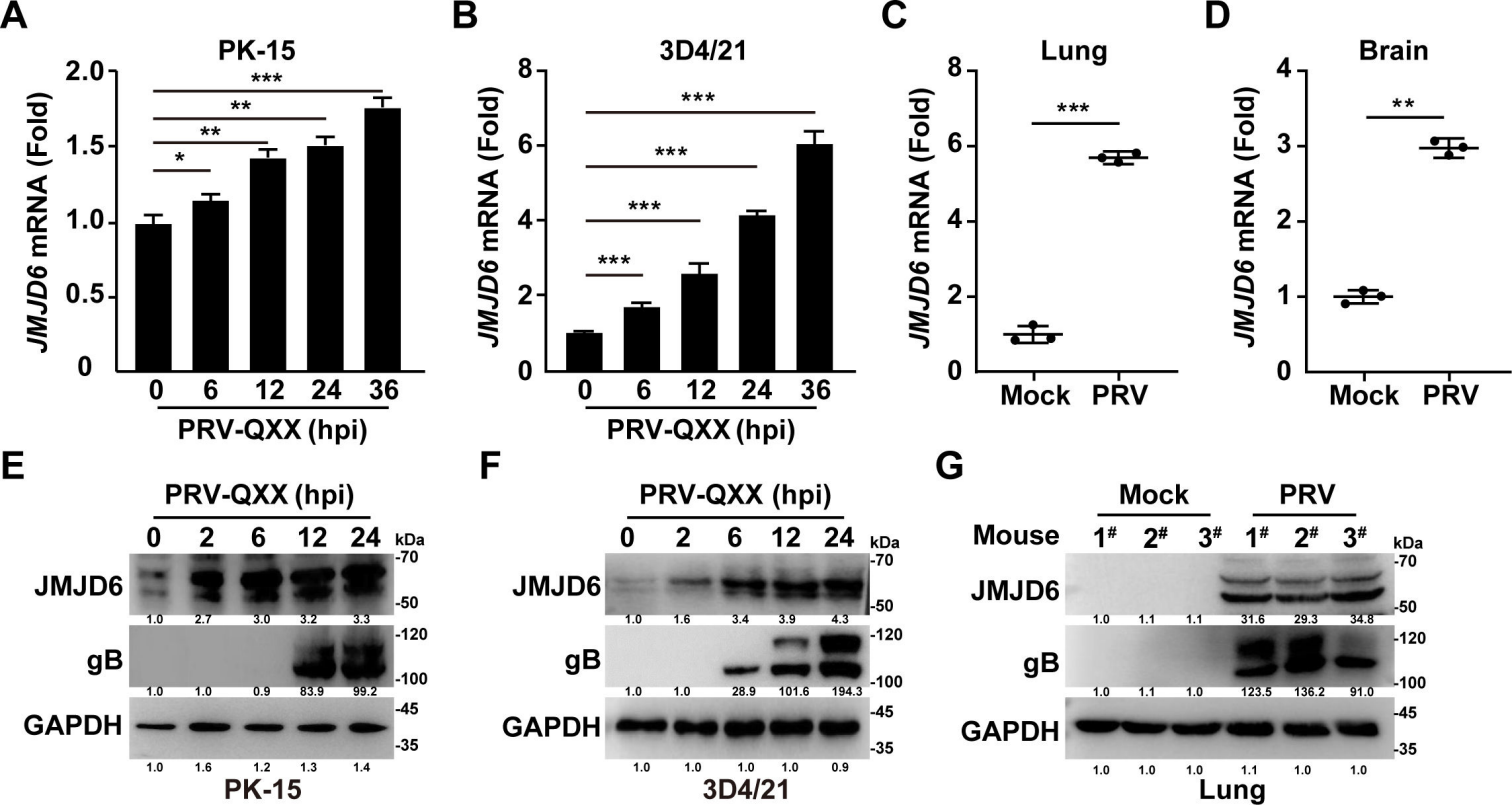
PRV infection stimulates JMJD6 expression. (**A and B**) PK-15 (**A**) and 3D4/21 cells (**B**) were infected with PRV-QXX (MOI = 0.1) for 0–36 h. The mRNA levels of JMJD6 were analyzed by qRT-PCR. *P < 0.05, **P < 0.01, ***P < 0.001; *n* = 3. (**C and D**) Female BALB/c mice were intranasally infected with PRV-QXX (5 × 10^3^ TCID_50_ per mouse, *n* = 3) for 3 days. The mRNA levels of JMJD6 in the lung (**C**) and brain (**D**) were analyzed by qRT-PCR. **P < 0.01, ***P < 0.001. (**E and F**) PK-15 (**E**) and 3D4/21 cells (**F**) were infected with PRV-QXX (MOI = 1) for 0–24 h. The protein levels of JMJD6 were analyzed by immunoblotting (*n* = 3). (**G**) Mice were treated as in panel C. The protein levels of JMJD6 in the lung were analyzed by immunoblotting (*n* = 3).

To investigate whether JMJD6 expression is affected by PRV infection, PK-15 and 3D4/21 cells were infected with PRV-QXX for periods ranging from 0 to 36 h. Quantitative real-time PCR (qRT-PCR) analysis revealed a time-dependent increase in *JMJD6* transcript levels in both cell lines (Fig. 2A and B). Similarly, intranasal infection of BALB/c mice with PRV-QXX resulted in elevated JMJD6 mRNA expression in both lung and brain tissues (Fig. 2C and D). We also assessed JMJD6 protein levels during infection. In PRV-infected PK-15 and 3D4/21 cells, JMJD6 protein expression progressively increased, correlating with rising levels of the viral glycoprotein gB (Fig. 2E and F). Consistent with the cellular results, JMJD6 protein was upregulated in the lungs of PRV-infected mice compared to mock-infected controls (Fig. 2G). Collectively, these findings demonstrate that PRV infection effectively induces JMJD6 expression both *in vitro* and *in vivo*.

### JMJD6 facilitates PRV replication

**Fig 3:**
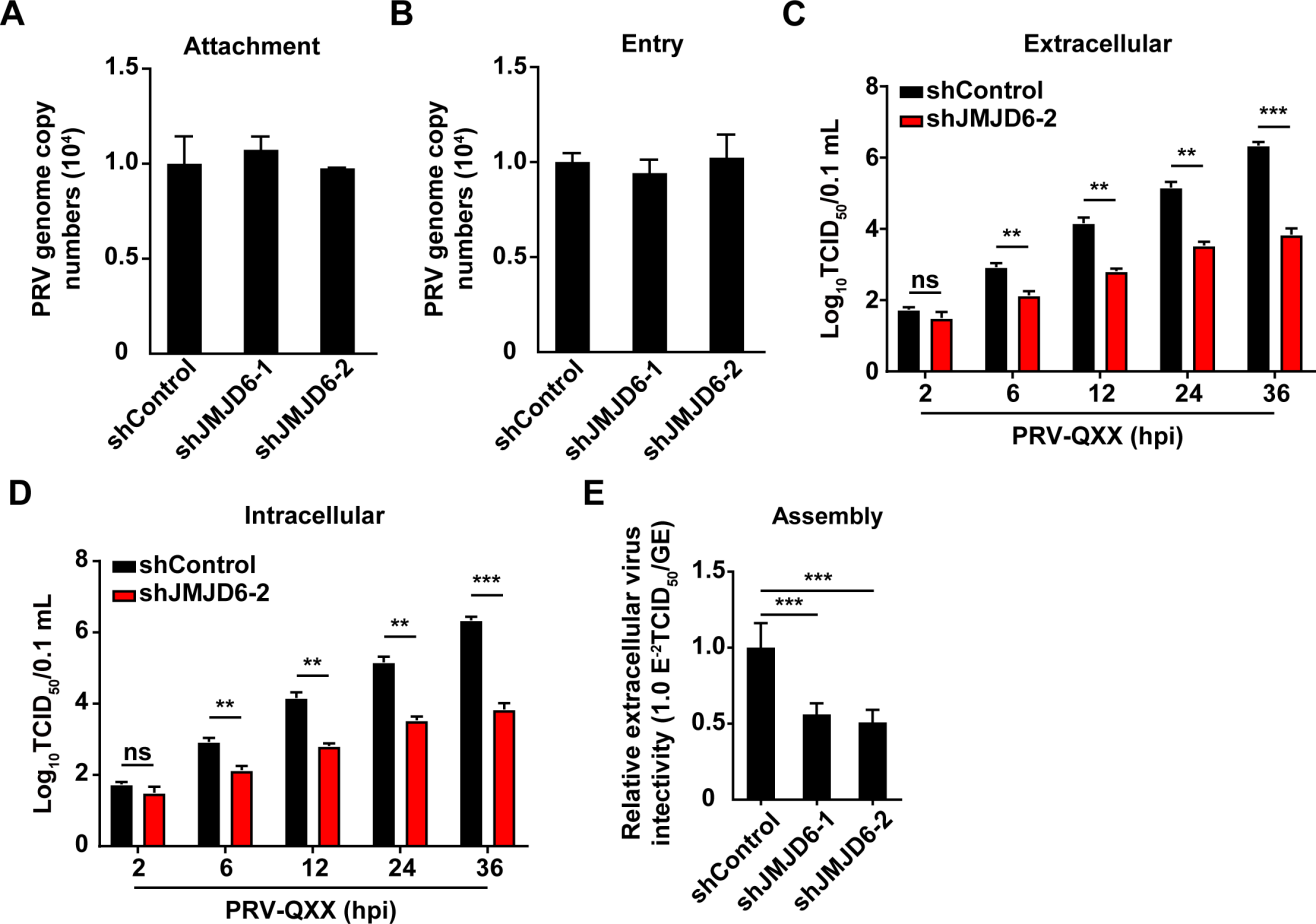
JMJD6 participates in PRV replication. (**A**) shControl, shJMJD6-1, and shJMJD6-2 PK-15 cells were incubated with PRV-QXX (MOI = 10) at 4°C for 2 h. After the cells were washed three times with ice-cold PBS, viral attachment was assessed by qRT-PCR analysis of genome copy numbers on the cell membrane (*n* = 3). (**B**) shControl, shJMJD6-1, and shJMJD6-2 PK-15 cells were incubated with PRV (MOI = 10) at 4°C for 2 h, extensively washed with ice-cold PBS three times, and then incubated at 37°C for 10 min to allow entry. After washing with trypsin (1 mg/mL) to remove residual virions on the PM, viral entry was detected by qRT-PCR analysis of viral genome copy numbers in the cells (*n* = 3). (**C and D**) Extracellular (**C**) and intracellular (**D**) viruses were collected from shControl and shJMJD6-2 PK-15 cells infected with PRV-QXX (MOI = 5) for 2–36 h. Viral titers were determined using the TCID_50_ assay. ns, no significance *P* > 0.05,**P < 0.01, ***P < 0.001; *n* = 3. (**E**) shControl and shJMJD6-2 PK-15 cells infected with PRV-QXX (MOI = 5) for 24 h. The efficiency of viral assembly in the supernatants was determined by comparing the infectious titers (TCID_50_ per milliliter) with the total number of PRV genome equivalent (GE). ***P < 0.001, *n* = 3.

To elucidate the mechanism by which JMJD6 facilitates PRV infection, we performed a series of functional assays. shControl, shJMJD6-1, and shJMJD6-2 PK-15 cells were incubated with PRV-QXX at 4°C for 2 h to allow viral attachment. qRT-PCR analysis of viral genome copy numbers associated with the plasma membrane revealed that *JMJD6* knockdown did not impair viral attachment (Fig. 3A). Viral entry was subsequently assessed by quantifying internalized viral genomes via qRT-PCR, and no significant difference was observed between *JMJD6*-knockdown and control cells (Fig. 3B). Notably, both shJMJD6-1 and shJMJD6-2 achieve robust *JMJD6* knockdown and produce comparable, statistically significant suppression of viral replication, confirming on-target specificity and phenotypic reproducibility across independent shRNA clones. To ensure maximal experimental rigor and biological relevance, we selected shJMJD6-2 as the primary model for downstream replication and release assays. As shown in Fig. 3C and D, intracellular and extracellular PRV titers in shJMJD6-2 cells were significantly reduced relative to shControl cells, providing consistent functional validation of JMJD6’s essential role in productive PRV infection. Moreover, virus release assays demonstrated that the efficiency of progeny virion release from shJMJD6-2 cells was markedly reduced compared to the control group (Fig. 3E). Collectively, these results indicate that JMJD6 promotes PRV infection by enhancing the replication and release of progeny virions, rather than by affecting viral attachment or entry.

### JMJD6 dampens H4K16 acetylation and innate antiviral signaling

**Fig 4:**
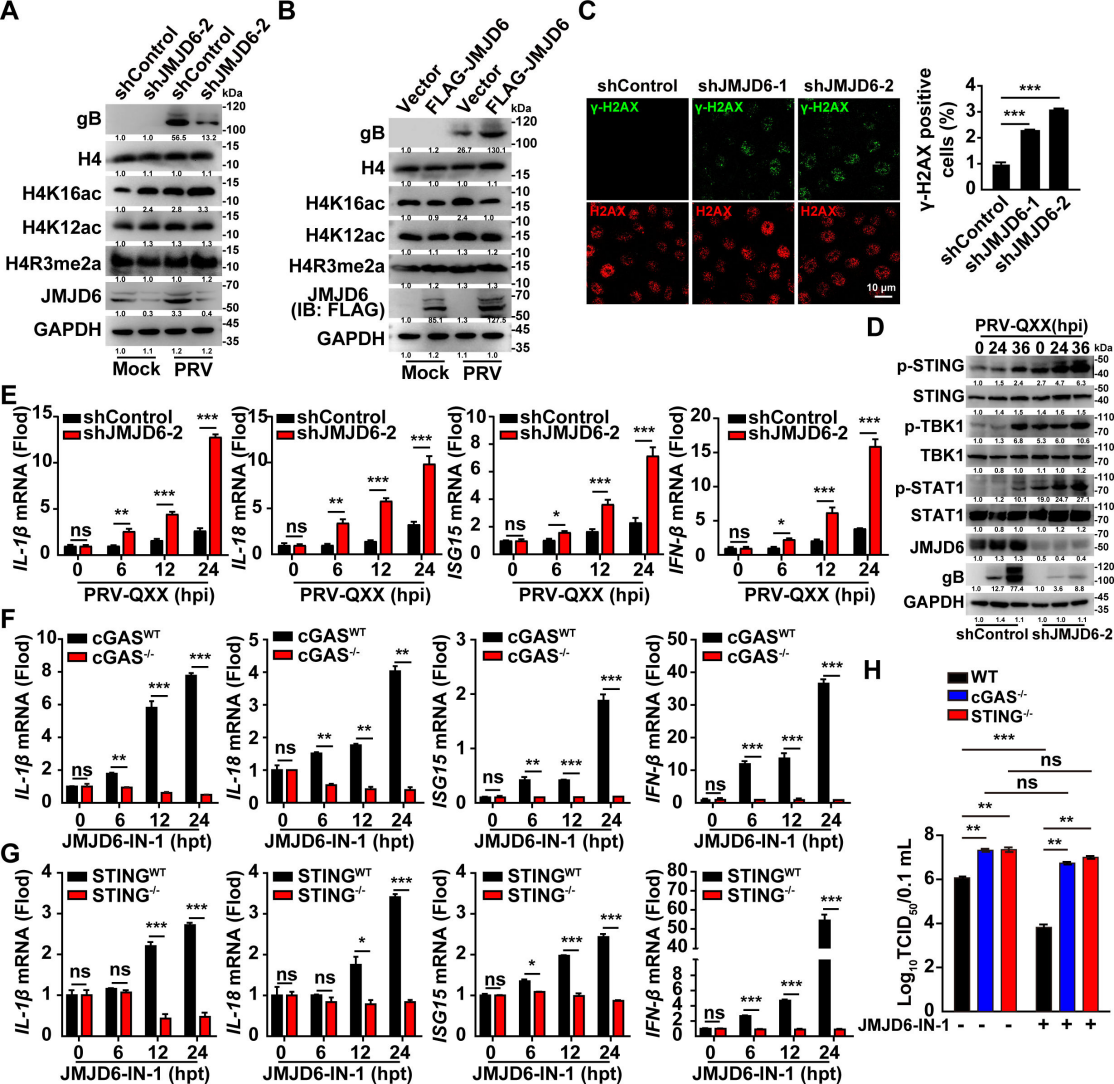
JMJD6 attenuates PRV-induced H4K16 acetylation and subsequent innate immune activation. (**A**) shControl and shJMJD6-2 PK-15 cells were mock-infected or infected with PRV-QXX (MOI = 1) for 24 h. The protein levels of H4, H4K16ac, H4K12ac, and H4R3me2a were analyzed by immunoblotting (*n* = 3). (**B**) PK-15 cells were transfected with empty vector or FLAG-JMJD6 for 24 h. Then, cells were mock-infected or infected with PRV-QXX (MOI = 1) for another 24 h. The protein levels of H4, H4K16ac, H4K12ac, and H4R3me2a were analyzed by immunoblotting (*n* = 3). (**C**) Immunofluorescence analysis of γ-H2AX and H2AX in shControl, shJMJD6-1, and shJMJD6-2 PK-15 cells (*n* = 30). ***P < 0.001, *n* = 3. (**D**) shControl and shJMJD6-2 PK-15 cells were infected with PRV-QXX (MOI = 0.1) for 0–36 h. The protein levels of STING, p-STING, TBK1, p-TBK1, STAT1, and p-STAT1 were analyzed by immunoblotting (*n* = 3). (**E**) shControl and shJMJD6-2 PK-15 cells were infected with PRV-QXX (MOI = 0.1) for 0–24 h. The mRNA levels of IL-1β, IL-18, ISG15, and IFN-β were analyzed by qRT-PCR. **P < 0.01, ***P < 0.001; *n* = 3. (**F**) cGAS^WT^ and cGAS^−/−^ PK-15 cells were treated with JMJD6-IN-1 (10 μM) for 0–24 h. The mRNA levels of IL-1β, IL-18, ISG15, and IFN-β were analyzed by qRT-PCR. **P < 0.01, ***P < 0.001; *n* = 3. (**G**) STING^WT^ and STING^−/−^ PK-15 cells were treated with JMJD6-IN-1 (10 μM) for 0–24 h. The mRNA levels of IL-1β, IL-18, ISG15, and IFN-β were analyzed by qRT-PCR. *P < 0.05, ***P < 0.001; *n* = 3. (**H**) WT, cGAS^−/−^ and STING^−/−^ PK-15 cells were treated with DMSO or JMJD6-IN-1 (10 μM) and infected with PRV-QXX (MOI = 1) for 24 h. The viral titers were analyzed by a TCID_50_ assay. **P < 0.001, *n* = 3. ns, no significance.

JMJD6 modulates diverse biological processes, including the regulation of chromatin architecture and the maintenance of genome integrity (20). Given that histone acetylation plays a critical role in safeguarding genomic stability (28), we investigated whether JMJD6 influences this modification. In mock-infected PK-15 cells, JMJD6 knockout increased H4K16 acetylation levels, whereas JMJD6 overexpression reduced them. In contrast, neither H4K12 acetylation nor H4R3 methylation was altered (Fig. 4A and B). Following PRV infection, elevated H4K16 acetylation levels were observed in both shControl and shJMJD6-2 PK-15 cells; however, cells overexpressing JMJD6 exhibited lower H4K16 acetylation levels compared to control cells (Fig. 4A and B). Furthermore, immunofluorescence analysis revealed a stronger γ-H2AX signal in JMJD6-knockdown cells than in controls (Fig. 4C), indicative of higher levels of DNA damage. These results suggest that JMJD6 suppresses H4K16 acetylation and attenuates DNA damage.

DNA damage is known to activate signaling pathways associated with innate immunity (29). Immunoblotting analysis demonstrated that PRV infection induced increased phosphorylation of STING, TBK1, and STAT1 in shControl PK-15 cells, with these effects being further enhanced in shJMJD6-2 PK-15 cells, indicating enhanced activation of the innate immune response (Fig. 4D). To further evaluate the inflammatory response, we assessed the transcriptional levels of key cytokines and interferon-stimulated genes using qRT-PCR. As shown in Fig. 4E, mRNA expression levels of *IL-1β*, *IL-18*, *ISG15*, and *IFN-β* were significantly elevated in shJMJD6-2 cells relative to control cells.

Given the central role of the cGAS–STING pathway in mediating immune and inflammatory responses to cytosolic DNA (30), we investigated its functional contribution in this context. Genetic ablation of cGAS and STING abolished the upregulation of *IL-1β*, *IL-18*, *ISG15*, and *IFN-β* transcription induced by the JMJD6 inhibitor JMJD6-IN-1 (Fig. 4F and G). Furthermore, the inhibitory effect of JMJD6-IN-1 on viral titer was abolished in PK-15 cells lacking *cGAS* or *STING* (Fig. 4H). These results demonstrate that JMJD6-mediated H4K16 hypoacetylation attenuates virus-triggered innate immune signaling.

### JMJD6 interacts with METTL23 in the nucleus

**Fig 5:**
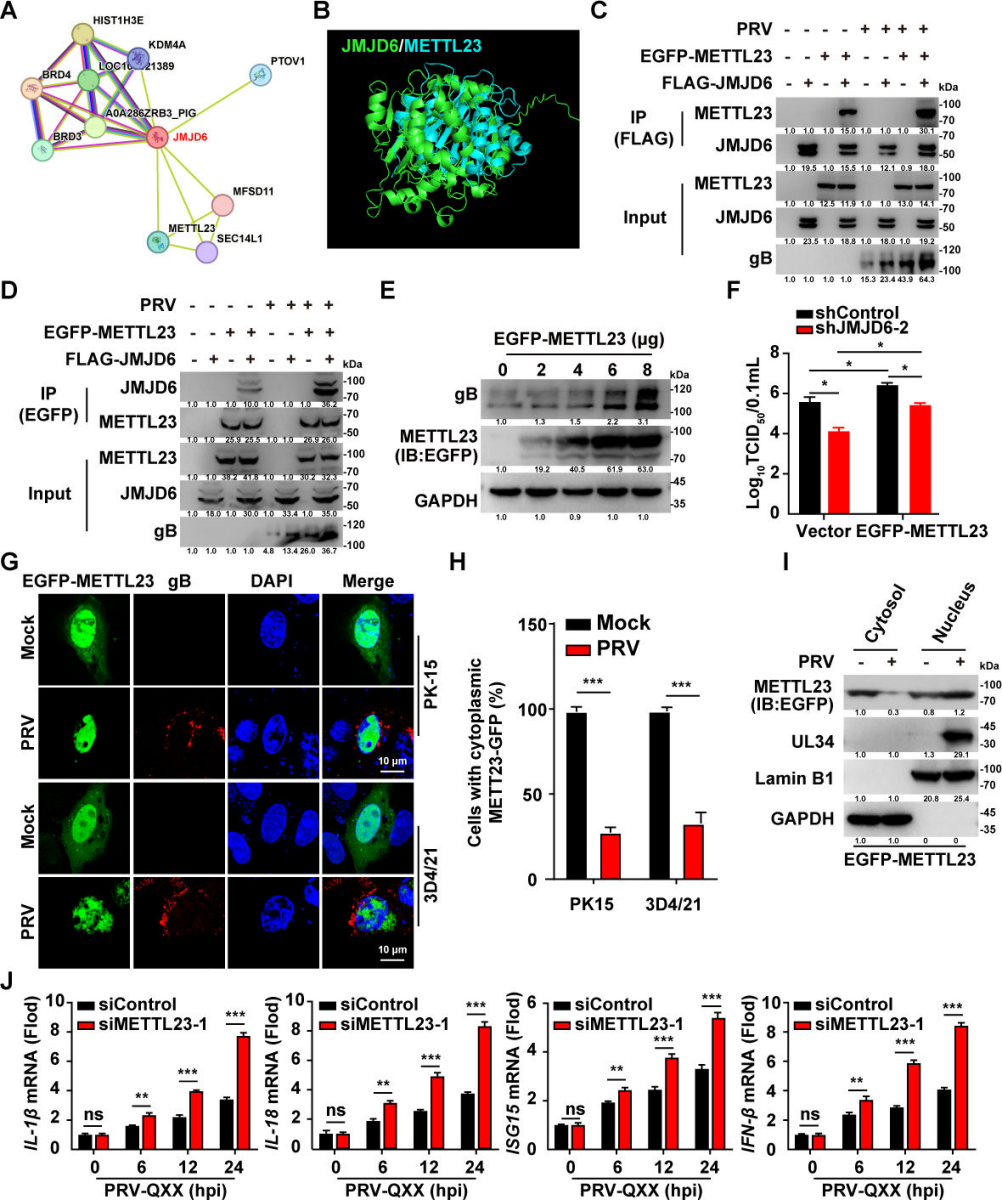
PRV infection facilitates the interaction between JMJD6 and METTL23 in the nucleus. (**A**) Prediction of JMJD6-interacting proteins using the STRING Interaction Network. (**B**) Prediction of the interaction between JMJD6 and METTL23 by AlphaFold. (**C**) PK-15 cells were either transfected with FLAG-JMJD6 and EGFP-METTL23 or co-transfected with FLAG-JMJD6 and EGFP-METTL23 for 24 h. Then, cells were mock-infected or infected with PRV-QXX (MOI = 1) for another 24 h. The interaction between JMJD6 and METTL23 was analyzed by Co-IP assay (*n* = 3). (**D**) PK-15 cells were either transfected with FLAG-JMJD6 and EGFP-METTL23 or co-transfected with FLAG-JMJD6 and EGFP-METTL23 for 24 h. Then, cells were mock-infected or infected with PRV-QXX (MOI = 1) for another 24 h. The interaction between JMJD6 and METTL23 was analyzed by Co-IP assay (*n* = 3). (**E**) PK-15 cells were transfected with EGFP-METTL23 (0–8 μg) for 24 h. Then, cells were infected with PRV-QXX (MOI = 1) for another 24 h. The protein levels of PRV gB were analyzed by immunoblotting (*n* = 3). (**F**) shControl and shJMJD6-2 PK-15 cells were transfected with empty vector or EGFP-METTL23 for 24 h. Then, cells were mock-infected or infected with PRV-QXX (MOI = 1) for another 24 h. The viral titers were analyzed by a TCID_50_ assay. *P < 0.05. (**G and H**) PK-15 and 3D4/21 cells were transfected with EGFP-METTL23 for 24 h. Then, cells were mock-infected or infected with PRV-QXX (MOI = 1) for another 24 h. The subcellular localization of EGFP-METTL23 was detected by fluorescence microscope (**G**). (**H**) The statistical results of the cells with nuclear localization in panel G (*n* = 50; a cell is scored as “cytoplasmic signal positive” if fluorescence is clearly detectable in both the cytoplasm and nucleus; it is scored as “cytoplasmic signal negative” if fluorescence is exclusively nuclear with no discernible signal in the cytoplasm). (**I**) PK-15 cells were transfected with EGFP-METTL23 for 24 h. Then, cells were mock-infected or infected with PRV-QXX (MOI = 1) for another 24 h. EGFP-METTL23 was detected with immunoblotting analysis in the cytosol and nuclear fraction (*n* = 3). (**J**) PK-15 cells were transfected with siControl or siMETTL23 for 24 h. Then, cells were infected with PRV-QXX (MOI = 1) for 0–24 h. The mRNA levels of IL-1β, IL-18, ISG15, and IFN-β were analyzed by qRT-PCR. **P < 0.01, ***P < 0.001; *n* = 3. ns, no significance.

To further investigate the mechanism by which JMJD6 facilitates PRV infection, we utilized the STRING Interaction Network to identify potential protein interaction partners of JMJD6 (Fig. 5A). Subsequently, the predicted interactions were evaluated using AlphaFold-based structural modeling, which revealed a potential binding interface between JMJD6 and METTL23 (Fig. 5B). Co-immunoprecipitation (Co-IP) assays confirmed the interaction between JMJD6 and METTL23, and this association was enhanced upon PRV infection (Fig. 5C and D).

We next evaluated the functional role of METTL23 during PRV infection. Transient transfection of an EGFP-METTL23 plasmid into PK-15 cells resulted in a dose-dependent increase in PRV gB expression (Fig. 5E). Consistently, viral titers were significantly elevated in EGFP-METTL23-expressing cells compared to control cells (Fig. 5F). Although *JMJD6* knockdown reduced viral production, overexpression of METTL23 markedly enhanced viral titers even under these conditions (Fig. 5F), suggesting that METTL23 promotes PRV infection in a pathway that is partially dependent on JMJD6.

Notably, METTL23 was localized in both the cytoplasm and nucleus of PK-15 and 3D4/21 cells under basal conditions. However, upon PRV infection, METTL23 accumulated predominantly in the nucleus (Fig. 5G and H). This infection-induced nuclear translocation was further confirmed by subcellular fractionation assays (Fig. 5I). Moreover, *METTL23* knockdown significantly enhanced the transcription of IL-1β, IL-18, ISG15, and IFN-β in response to viral infection (Fig. 5J). Collectively, these findings indicate that METTL23 cooperates with JMJD6 in the nucleus to facilitate PRV replication.

### Inhibition of JMJD6 attenuates PRV infection in mice

**Fig 6:**
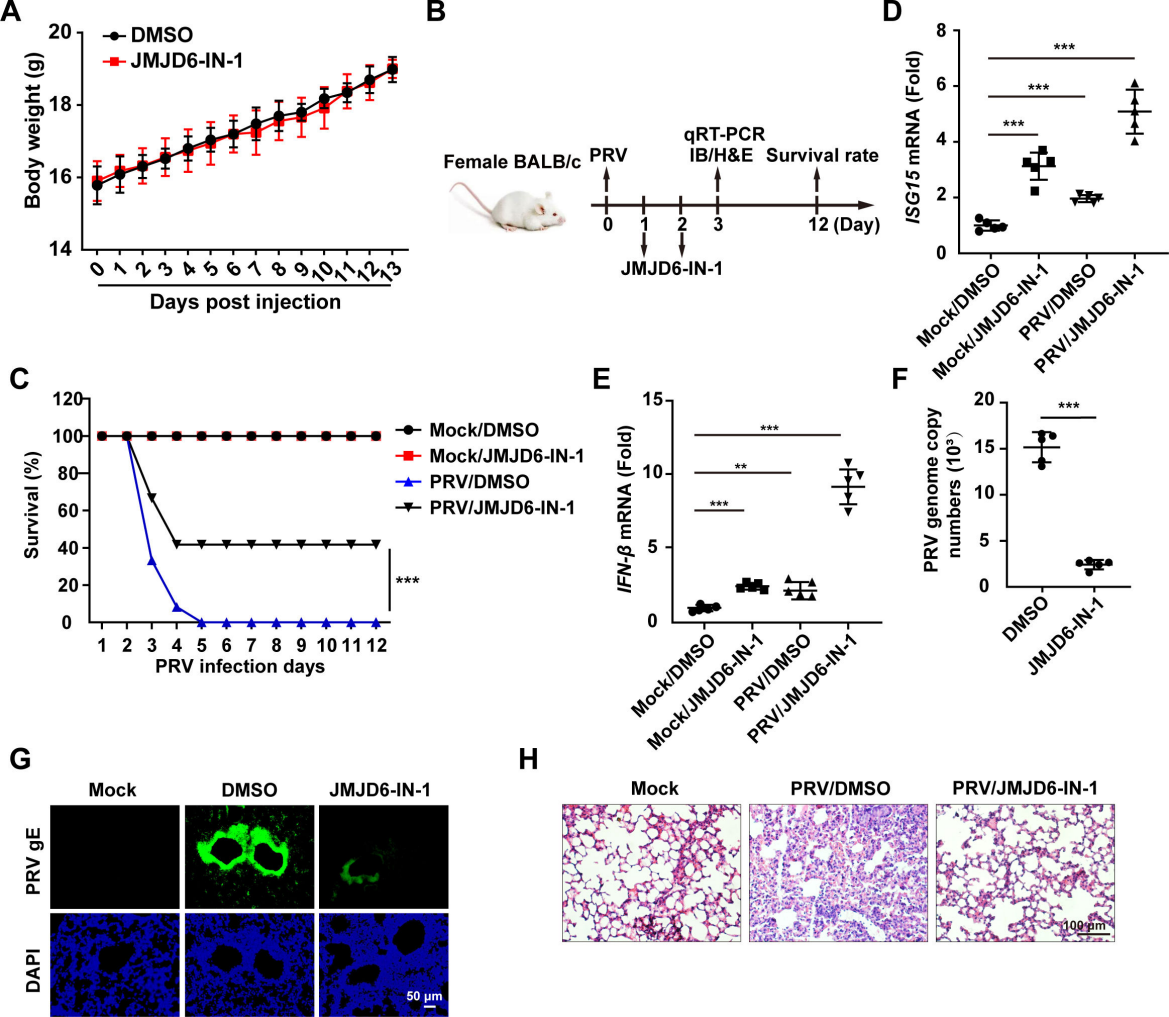
Inhibition of JMJD6 restricts PRV infection in mice. (**A**) Female 8-week-old mice (*n* = 5 per group) were injected S.C. daily for 13 days with 100 μL DMSO or JMJD6-IN-1 at 3 mg/kg in 100 μL DMSO. Daily average weights of the mice in each group are shown. (**B**) Schematic diagram of the experimental procedure for PRV-QXX challenge and JMJD6-IN-1 treatment in mice. (**C**) On day 0, mice were mock-infected or intranasally infected with PRV-QXX (5 × 10^3^ TCID_50_ per mouse). On days 2 and 3, mice were injected with DMSO or JMJD6-IN-1 (3 mg/kg). The survival rate was monitored daily for 12 days (*n* = 12). (**D and E**) The mRNA levels of ISG15 (**D**) and IFN-β (**E**) in the lung of mice treated as in panel B were analyzed by qRT-PCR. **P < 0.01, ***P < 0.001 (*n* = 5). (**F**) On day 0, mice were intranasally infected with PRV-QXX (5 × 10^3^ TCID_50_ per mouse). On days 2 and 3, mice were injected with DMSO or JMJD6-IN-1 (3 mg/kg). On day 3, viral genome copy numbers in the lung were analyzed by qRT-PCR. ***P < 0.001 (*n* = 5). (**G**) PRV gE expression in the lung of mice treated as in panel E were examined by immunohistochemistry. (**H**) Lung sections from mice treated as in panel E were stained by H&E.

To evaluate the role of JMJD6 in PRV proliferation *in vivo*, a systematic assessment of the potential toxicity of JMJD6-IN-1 was first performed. Based on body weight monitoring, administration of JMJD6-IN-1 at a dose of 3 mg/kg did not adversely affect mouse growth, and no mortality was observed following exposure to this dose (Fig. 6A). Subsequently, the therapeutic efficacy of the JMJD6 inhibitor JMJD6-IN-1 was evaluated in a murine model of PRV infection (Fig. 6B). Mice treated with JMJD6-IN-1 exhibited a significant reduction in mortality following PRV challenge compared to control animals (Fig. 6C). qRT-PCR analysis of lung tissues revealed that JMJD6-IN-1 treatment enhanced the mRNA expression levels of *ISG15* and *IFN-β* post-PRV infection (Fig. 6D and E). Furthermore, both viral load and gE expression in the lungs were markedly reduced in JMJD6-IN-1-treated mice relative to DMSO-treated controls (Fig. 6F and G). Histopathological examination further demonstrated attenuated lung injury in JMJD6-IN-1-administered mice compared to those receiving DMSO (Fig. 6H). These findings collectively indicate that pharmacological inhibition of JMJD6 ameliorates PRV infection *in vivo*.

## DISCUSSION

Our findings demonstrate that JMJD6 is significantly upregulated during PRV infection and functions as an epigenetic regulator facilitating virion release. Mechanistically, JMJD6 promotes chromatin condensation through selective suppression of H4K16 acetylation, thereby attenuating DNA damage signaling and preventing aberrant activation of the cGAS–STING pathway and subsequent IFN production, ultimately creating a cellular environment conducive to viral replication. In contrast, depletion of JMJD6 triggers DNA damage accumulation, increases H4K16 acetylation, impairs chromatin condensation, and robustly activates the IFNs’ response, leading to effective suppression of viral replication. Notably, pharmacological inhibition of JMJD6 alone is sufficient to restore interferon signaling *in vivo* and significantly reduce viral titers, underscoring its promise as a therapeutic target for herpesvirus infections.

Our observation that exogenous JMJD6 overexpression further enhances viral replication, despite its endogenous upregulation during infection, raises the question of how such additional expression can still confer a phenotype. While the precise mechanism remains to be elucidated, two non-mutually exclusive possibilities may explain this effect. First, exogenous overexpression driven by the strong CMV promoter likely results in supraphysiological JMJD6 protein levels that exceed the peak concentrations achieved by infection-induced endogenous expression, thereby further augmenting its proviral function beyond a saturation point. Second, the timing of expression may be critical: exogenous JMJD6 is already present at high levels prior to infection, whereas endogenous JMJD6 induction occurs only after infection has initiated, potentially providing a functional advantage during an early window when JMJD6-mediated chromatin modulation and DNA damage signaling are most limiting for viral replication. This temporal distinction aligns with our finding that JMJD6 acts at the level of chromatin condensation, a process operative from the earliest stages of viral entry and genome delivery. Future studies employing inducible expression systems or precise titration of JMJD6 levels could help distinguish between these possibilities.

H4K16 hypoacetylation has previously been associated with transcriptional repression and chromatin compaction during yeast and mammalian development (31, 32), but its role in antiviral defense remains largely unexplored. Our findings are consistent with recent reports demonstrating that pharmacological hyperacetylation of H4K16 enhances STING-dependent IFN responses. However, they contrast with studies attributing a proviral function to H4K16 acetylation during influenza virus replication (33). Notably, JMJD6 is traditionally characterized as a transcriptional co-activator capable of demethylating H3R2 or H4R3 (19); here, we identify a previously unrecognized non-histone substrate through which JMJD6 recruits HDAC-containing complexes to mediate H4K16 deacetylation. This context-dependent substrate switch may account for the dual regulatory role of JMJD6 as either a transcriptional enhancer or repressor, depending on cellular conditions (34).

The successful navigation of chromatin’s complex and dynamic architecture is a critical prerequisite for an efficient viral infection cycle (35). We further uncovered a functional interplay between the histone demethylase JMJD6 and the protein methyltransferase METTL23. METTL23 functions as a histone modifier, targeting histone H3 at arginine 17 for asymmetric dimethylation, while JMJD6 modulates H4K16 acetylation and DNA damage signaling, as demonstrated in this study (36). Notably, our observation that METTL23 overexpression retains the ability to enhance viral replication even when JMJD6 is depleted suggests that these two factors do not function exclusively as an obligate complex within a single linear pathway. Instead, they likely act through convergent but separable mechanisms to promote PRV replication. This interpretation is consistent with their distinct molecular activities: JMJD6 primarily influences chromatin condensation and the cGAS–STING pathway via H4K16ac modulation, whereas METTL23 catalyzes H3R17 methylation and may independently regulate NF-κB activity (37). These parallel contributions could collectively create a cellular environment conducive to viral propagation. While their physical interaction may facilitate optimal functional synergy, each protein retains the capacity to exert proviral effects in the absence of the other. The precise molecular interface through which these pathways converge—and whether their interplay is sensitive to cellular metabolic flux or infection stage—remains to be fully elucidated. Deciphering these questions will be crucial for understanding how herpesviruses exploit multiple host protein-modifying enzymes to coordinately evade immune surveillance and remodel chromatin.

Several limitations and future research directions merit emphasis. First, our conclusions are based on a single PRV strain and immortalized porcine cell lines (38, 39); whether clinical isolates or other alphaherpesviruses utilize similar JMJD6-METTL23-dependent mechanisms requires further validation. Second, although our findings suggest that JMJD6 modulates H4K16ac, its direct enzymatic activity on histone substrates has not been confirmed *in vitro*, leaving open the possibility of indirect regulation through intermediary factors. Third, regarding the *in vivo* efficacy of JMJD6-IN-1, we acknowledge that our toxicity assessment was limited to body weight monitoring and general behavioral observations (e.g., fur condition, activity level, and food intake), which showed no notable differences between inhibitor-treated and control groups. However, body weight alone is an insensitive measure for comprehensive toxicity evaluation, and detailed pharmacokinetic/pharmacodynamic (PK/PD) studies were not performed in this study. We note that JMJD6-IN-1 (also referred to as SKLB325 in some studies) has been previously characterized in cancer models, with reported IC_50_ values in the low micromolar range and demonstrated *in vivo* efficacy at doses comparable to ours without overt toxicity (40, 41). These published data provide indirect support for the dosage selection in our study. Nevertheless, we recognize that systematic toxicological profiling and PK/PD analysis are necessary to fully evaluate the therapeutic potential and safety of JMJD6 inhibitors for herpesvirus infections. Future studies should include comprehensive assessments of drug metabolism, tissue distribution, and potential off-target effects, as well as evaluation of whether dual targeting of JMJD6 and METTL23 synergistically enhances antiviral responses without triggering autoinflammatory pathology.

## MATERIALS AND METHODS

### Mice

Female BALB/c mice (6–8 weeks old) were housed under specific-pathogen-free conditions with a controlled 12-h light–dark cycle and temperature (22°C). For the survival study, animals were assigned to four groups: Mock/DMSO, Mock/JMJD6-IN-1, PRV/DMSO, and PRV/JMJD6-IN-1 (*n* = 12 per group). Mice were monitored twice daily for clinical signs throughout the experiment. The predefined euthanasia criteria included (i) body weight loss exceeding 20% of the initial body weight; (ii) severe neurological signs such as hind limb paralysis, ataxia, or seizures; (iii) moribund state defined as prostration (inability to move after gentle prodding), hypothermia, or prolonged unresponsiveness to stimuli. Mice meeting any of these criteria were considered to have reached the experimental endpoint and were immediately euthanized using pentobarbital sodium (90 mg/kg, intraperitoneal injection). these animals were included in the survival analysis as mortality events. No exceptions to these criteria were applied. Sample processing for qRT-PCR and histology was performed in a blinded manner.

### Cells and viruses

PK-15, 3D4/21, and HEK293T cells were maintained under standard culture conditions in Dulbecco’s Modified Eagle Medium (DMEM) supplemented with 10% fetal bovine serum (FBS), 1% penicillin/streptomycin, and 1% L-glutamine at 37°C in a humidified atmosphere containing 5% CO_2_. *cGAS*^−/−^ and *STING*^−/−^ PK-15 cell lines were generated and used as previously described (42).

To generate stable knockdown cell lines, HEK293T cells were co-transfected with lentiviral packaging plasmids (psPAX2 and pMD2.G) alongside either a scrambled control shRNA or one of the following gene-specific shRNA constructs: shControl, 5′-GCCACAACGTCTATATCATGG-3′; shJMJD6-1, 5′-GCGGTATGAAAGACCTTACA-3′; and shJMJD6-2, 5′-CGATGGCTACTCAGTGAAGA-3′. The culture medium was replaced 6 h post-transfection. Lentiviral particles were harvested 48 h thereafter and used to infect the parental cell lines. Stable transductants were selected via puromycin treatment.

The virulent PRV strain QXX (PRV-QXX) was generously provided by Prof. Yong-Tao Li from the College of Veterinary Medicine at Henan Agricultural University (43).

### Chemicals and antibodies

JMJD6-IN-1 (HY-139434) and protease inhibitor cocktail (HY-K0021) were purchased from MedChemExpress. Antibodies against FLAG (66008-4-Ig), H4R3me2a (39006), GAPDH (60004-1-Ig), and EGFP (66002-1-Ig) were purchased from Proteintech; anti-JMJD6 (bs-6842R) was acquired from Bioss; antibodies against γ-H2AX (#80,312), H2AX (#7631), H4 (13919), H4K16ac (13534), H4K12ac (13944), p-STING (72971), p-TBK1 (5483), p-STAT1 (9167), and Lamin B1 (17416) were purchased from Cell Signaling Technology. Antisera specific to PRV gB and UL34 were generated by immunizing mice with purified recombinant gE and UL34 proteins.

### Plasmid construction and transfection

The coding sequences of *JMJD6* and *METTL23* were amplified from the cDNA of PK-15 cells and cloned into p3×Flag-CMV-14 or pEGFP-N1 to generate FLAG-JMJD6 and EGFP-METTL23. The JMJD6 mutants H187A and D189A were generated by site-directed mutagenesis following the manufacturer’s protocol (Stratagene, 200519). Transient transfection of these expression plasmids was carried out using TurboFect reagent (Thermo Fisher Scientific, R0531) according to the manufacturer’s instructions.

### Cell viability analysis

Cell viability was quantified using the Cell Counting Kit-8 (CCK-8; Biodragon, BDXB0002). shControl, shJMJD6-1, and shJMJD6-2 PK-15 cells were plated in 96-well plates for 0–36 h. According to the manufacturer’s instructions, 10 µL of the CCK-8 reagent was added to each well, and the plates were incubated for 2 h. Absorbance was then read at 450 nm to determine viability.

### Cytosol and nuclear protein fractionation

According to the manufacturer’s instructions, nuclear and cytoplasmic extraction was performed with NE‐PER Nuclear and Cytoplasmic Extraction Reagents (Thermo Fisher Scientific, 78,833). The extracted fractions were subjected to immunoblotting analysis.

### Immunoblotting analysis

Cells were harvested and lysed in a lysis buffer containing 50 mM Tris–HCl (pH 8.0), 150 mM NaCl, 1% Triton X-100, 1% sodium deoxycholate, 0.1% SDS, and 2 mM MgCl_2_, supplemented with protease inhibitor cocktail. To analyze the post-translational modification of histones, the nuclear fraction was used. The resulting protein lysates were separated by SDS-PAGE and subsequently transferred onto a polyvinylidene difluoride membrane. After blocking with 5% non-fat milk for 1 h at room temperature, the membrane was incubated with primary antibodies overnight at 4°C. This was followed by incubation with horseradish peroxidase (HRP)-conjugated secondary antibodies for 1 h at room temperature. Protein signals were detected using Luminata Crescendo Western HRP Substrate (Millipore, WBLUR0500) and imaged using a GE AI600 imaging system.

### RNA extraction and qRT-PCR

Total RNA was extracted with TRIzol reagent (TaKaRa, 9108), and cDNA was synthesized using a PrimeScript RT reagent kit (TaKaRa, RR047A). qRT-PCR was then carried out in triplicate with SYBR Premix Ex Taq (TaKaRa, RR820A), following the manufacturer’s protocol. Gene expression levels were normalized to ACTB, and melting curve analysis confirmed amplification specificity. Relative expression changes were calculated using the 2^−ΔΔCt^ method. The primers used in this study are porcine *ACTB*-Fw: 5′-CTGAACCCCAAAGCCAACCGT-3′, *ACTB*-Rv: 5′-TTCTCCTTGATGTCCCGCACG-3′; porcine *JMJD6*-Fw: 5′-CAAGCGCTGGTGCTTATTCC-3′, *JMJD6*-Rv: 5′-TCCAGGGGTTTGAATTCGGG-3′; porcine *IL-1β*-Fw: 5′-CCATCCACTGAGCCAGCCTT-3′, *IL-1β*-Rv: 5′-TGCCAAGGACAGAGGACTGC-3′; porcine *IL-18*-Fw: 5′-ATGCCTGATTCTGACTGTTCAG-3′, *IL-18*-Rv: 5′-TATCATCATGTCCAGGAACACTTC-3′; porcine *ISG15*-Fw: 5′-ATGCCCCCTTGCCCTCTCCAGTG-3′, *ISG15*-Rv: 5′-TCCGATGCCATCATGCAGTCCCT-3′; porcine *IFN-β*-Fw: 5′-AGTTGCCTGGGACTCCTCAA-3′, *IFN-β*-Rv: 5′-CCTCAGGGACCTCAAAGTTCAT-3′; mouse *ACTB*-Fw: 5′-CCCCATTGAACATGGCATTG-3′, *ACTB*-Rv: 5′-ACGACCAGAGGCATACAGG-3′; mouse *JMJD6*-Fw: 5′-GACGTCTTGATCACCCGAT-3′, *JMJD6*-Rv: 5′-GCAGCAGAGTCACGGATGTA-3′; mouse *ISG15*-Fw: 5′-GGTAACGATTTCCTGGTGTC-3′, *ISG15*-Rv: 5′-CTTAAGCGTGTCTACAGTCTG-3′; and mouse *IFN-β*-Fw: 5′-ATGAGTGGTGGTTGCAGGC-3′, *IFN-β*-Rv: 5′-TGACCTTTCAAATGCAGTAGATTCA-3′.

### RNAi

The siRNAs (siControl, 5′-UUCUCCGAACGUGUCACGUTT-3′, and siMETTL23, 5′-GCAUCAAGUUCUCCGAGAATT-3′) were synthetized by GenePharma (Shanghai, China) and transfected into PK15 cells with Lipofectamine RNAiMAX (Invitrogen, 13778150), according to the manufacturer’s instructions.

### Immunofluorescence analysis

Cells grown on coverslips in 24-well plates were fixed with 4% (wt/vol) paraformaldehyde for 20 min at room temperature. Following three washes with phosphate-buffered saline (PBS), cells were permeabilized with 0.1% Triton X-100 for 10 min and blocked with 10% FBS in PBS. Primary antibodies, diluted in 10% FBS, were applied and incubated for 1 h at room temperature. After three additional PBS washes, cells were incubated with fluorescently labeled secondary antibodies—diluted in the same blocking solution—for 1 h at room temperature. Nuclei were counterstained with DAPI for 10 min, and coverslips were mounted using FluorSave Reagent (Millipore, 345789). Fluorescence images were acquired using a Zeiss LSM 800 laser scanning confocal microscope.

### Co-IP assay

Following a 24-h transfection with the indicated plasmids, cells were harvested and lysed in 1 mL of lysis buffer (PBS containing 1% NP-40, 5 mM EDTA, and 5 mM EGTA). The lysates were clarified by centrifugation at 16,000 × *g* for 10 min at 4°C. For immunoprecipitation, 900 μL aliquots of the supernatant were incubated with 40 μL of a 1:1 slurry of Sepharose beads conjugated to either control IgG (GE Healthcare, 17-0969-01), Anti-GFP Magnetic Beads (Beyotime, P2132), or anti-FLAG mouse monoclonal antibody (Sigma-Aldrich, A2220) for 4 h at 4°C. The beads were washed four times with lysis buffer, and bound proteins were eluted by boiling in SDS sample buffer (Solarbio, P1015) for 10 min prior to immunoblotting analysis.

### Viral attachment assay

To assess viral attachment, cells were incubated with PRV-QXX at 4°C for 2 h. Following three rigorous washes with ice-cold PBS, cell-associated viral particles were quantified by qRT-PCR analysis to determine PRV genomic copy numbers (43).

### Viral entry assay

To assess viral entry, cells were first incubated with PRV-QXX at 4°C for 2 h to allow viral attachment. Following three washes with ice-cold PBS, the temperature was shifted to 37°C for 10 min to initiate internalization. Residual non-internalized virions were removed by treatment with trypsin (1 mg/mL). Viral entry was then quantified by qRT-PCR analysis to determine PRV genomic copy numbers (43).

### TCID_50_ assay

On day 0, Vero cells were seeded in 96-well plates at a density of 1 × 10⁴ cells per well. On the following day (day 1), cells were inoculated with 10-fold serial dilutions of virus (ranging from 10⁻¹ to 10⁻¹²) for 1 h at 37°C. After incubation, the viral inoculum was removed by aspiration, and the cells were gently washed with PBS. Subsequently, 200 μL of maintenance medium (DMEM supplemented with 2% fetal bovine serum) was added to each well, and the cultures were incubated at 37°C under 5% CO₂ for 3–5 days. The development of cytopathic effect was monitored daily. The TCID_50_ was calculated using the Reed–Muench method.

### Histological analysis

Lung tissues dissected from mice were fixed in 4% (wt/vol) paraformaldehyde overnight, embedded in paraffin (Solarbio, YA0012), sectioned into 4–5 μm thick slices, and stained with hematoxylin (Sigma-Aldrich, MHS1) and eosin (Sigma-Aldrich, E4009) solutions.

### Statistical analysis

All statistical analyses were performed using Prism 7 software (GraphPad Software). For comparisons between the two groups, a two-tailed Student’s *t*-test was used, and a *P* value less than 0.05 was considered statistically significant. Survival data from mouse studies were analyzed by generating Kaplan–Meier curves with log-rank test assessment. For quantitative experiments, including qRT-PCR, TCID_50_, and CCK-8 assays, technical triplicates within each biological replicate were averaged, and these averaged values from at least three independent biological replicates (*n* = 3) were used for statistical analysis. All Western blot and immunofluorescence data were representative of at least three independent experiments; for quantifiable data, the displayed image corresponds to the replicate with signal intensity closest to the mean of all replicates.

## Data Availability

The data that support the findings of this study are openly available in Mendeley Data at https://data.mendeley.com/datasets/pch8nz49rc/1, doi:10.17632/pch8nz49rc.1.
